# Supplementary figures and images for: Transcriptome-wide Mendelian randomisation exploring dynamic CD4+ T cell gene expression in colorectal cancer development
Source: medRxiv. 2025 Apr 17:2025.04.15.25325863. Preprint. [Version 1] doi: 10.1101/2025.04.15.25325863 (PMC12047913; doi:10.1101/2025.04.15.25325863)

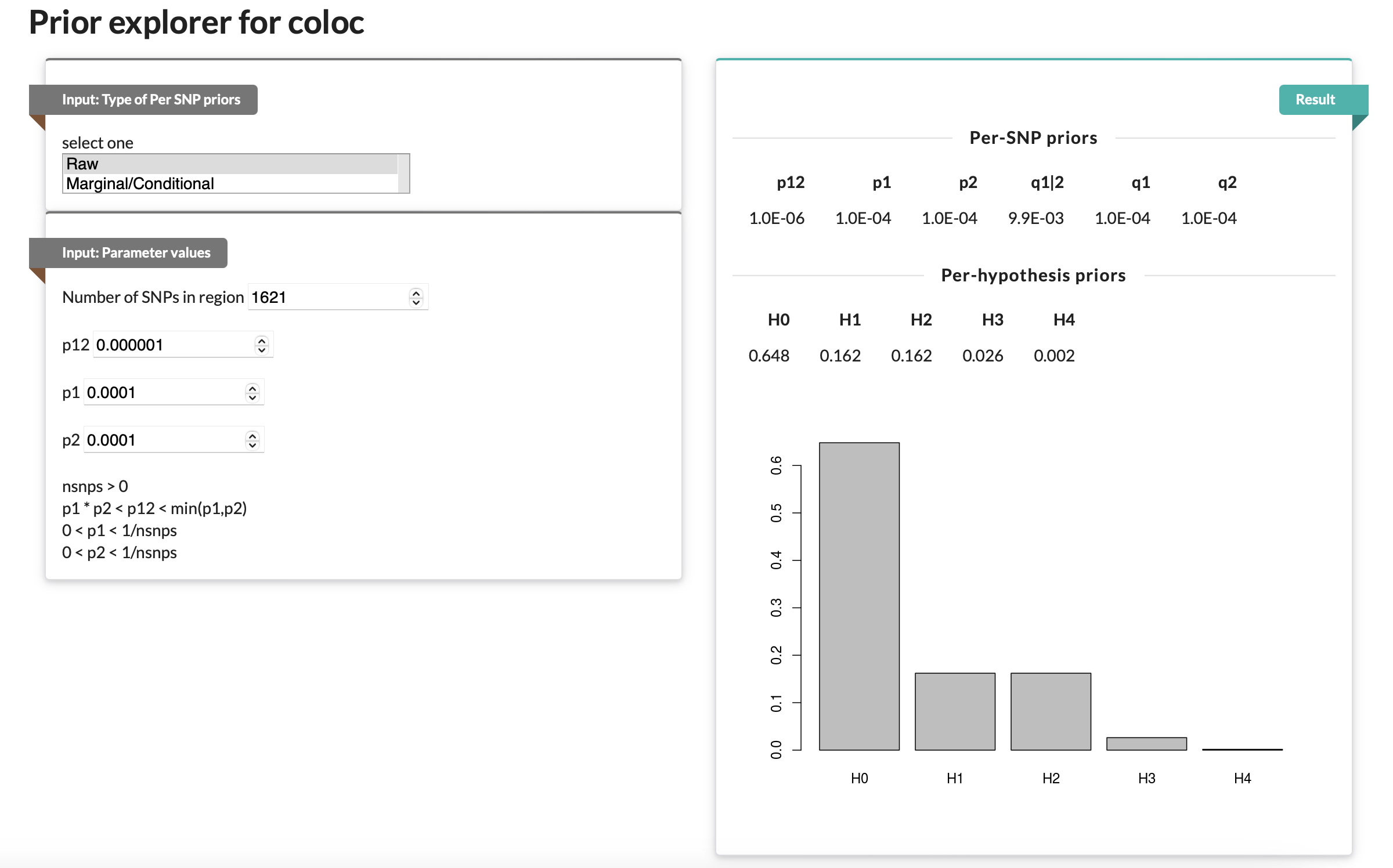

Supplement: Supplement 3 — Supplementary figure 1. Priors set for the genetic colocalisation analysis. [file media-3.jpg]
